# Analysis of factors associated with the umbilical cord pitch value by ultrasound measurement in late pregnancy

**DOI:** 10.1186/s12884-023-05894-x

**Published:** 2023-08-12

**Authors:** Chen Liang, Youfeng Xu

**Affiliations:** 1 Department of Ultrasound, The First Hospital of Ningbo University, Ningbo, Zhejiang 315000 China; 2 Department of Ultrasound, Ningbo Women and Children’s Hospital, Ningbo, Zhejiang 315000 China

**Keywords:** Prenatal ultrasound assessment, Pitch value of umbilical cord, Cord entanglement, Cord torsion, Cord edema

## Abstract

**Background:**

This study aimed to investigate the factors associated with the antenatal umbilical cord pitch value by ultrasound measurement in late pregnancy. We investigated the factors associated with the umbilical cord pitch value under prenatal ultrasound measurement.

**Methods:**

This study included 528 pregnant women who underwent routine antenatal ultrasound examinations in Ningbo Women and Children’s Hospital from December 2020 to August 2021. Their umbilical cord pitch values and diameter, Wharton’s jelly thickness, amniotic fluid indexes, umbilical artery blood flow parameters, and other relevant data, such as ages and gestational ages, were measured. Information about delivery methods, placenta, umbilical cord, and neonatal weight were recorded during follow-up. Statistical analysis was performed on the above data, and the factors associated with the pitch values were analyzed by linear regression.

**Results:**

This study revealed that cord torsion (*p* < 0.001, 95% confidence interval [CI]=-34.81 to -19.01), cord entanglement (*p* < 0.001, 95% CI = 10.71 to 20.11), thickening of Wharton’s jelly (*p* = 0.001, 95% CI = 5.39 to 20.24), and cord edema (*p* = 0.015, 95%CI = 2.09 to 19.44), gestational age (*p* = 0.024, 95%CI = 0.14 to 1.89), age of pregnant woman (*p* = 0.009, 95%CI= -1.15 to -0.16), and neonatal weight (*p* = 0.011, 95%CI = 0.002 to 0.012) were significantly correlated with the pitch values.

**Conclusion:**

The umbilical cord pitch value significantly correlated with cord entanglement, cord torsion, cord edema, Wharton’s jelly thickening, gestational age, age of the pregnant woman, and neonatal weight. Notably, the pitch value by prenatal ultrasound measurement is predictive of cord morphological abnormalities such as cord entanglement, cord torsion, cord edema, and Wharton’s jelly thickening.

## Background

The spiral structure is one of the important features of the umbilical cord. Prenatal ultrasonographic detection of the spiral structure of the umbilical cord has always been a difficult task, especially in late pregnancy, when fetal obscuration causes difficulty in full cord-length observation [1]. Debates in the medical community over the ultrasound detection of various umbilical cord spiral structures remain considerable, and as of yet, no international standard for spiral structures in prenatal ultrasonography has been created [1, 2]. Hence, a lot of high-quality studies remain expected. The umbilical cord pitch value is one of the indicators for quantitative spiral structure assessment by prenatal ultrasonography. The pitch value measured by prenatal ultrasonography in late pregnancy reflects the actual number of umbilical cord spirals, to some extent. However, the value may be affected by several factors in utero, and no literature has been retrieved to systematically analyze the factors. This study investigated the factors associated with umbilical cord pitch value under prenatal ultrasound measurement.

## Study subjects and methods

### Study subjects

This study included pregnant women in late pregnancy (from the 28th week of gestation to before delivery) who underwent routine prenatal ultrasound examinations in Ningbo Women and Children’s Hospital from December 2020 to August 2021. Women with normal pregnancies were categorized into the normal group while women with abnormal pregnancies were categorized into the abnormal group. The inclusion criteria of the pregnant women were as follows: women (with a routine obstetric examination, clear time of last menstruation, and accurate gestation ages) in the 28th week of gestation to before delivery; women with singleton pregnancy; women with no serious fetal malformation by prenatal ultrasound examination, umbilical cord consisting of two umbilical arteries, and one umbilical vein with at least two spirals in the displayable umbilical cord segments; women with complete clinical data and giving birth in the above hospital. Exclusion criteria include pregnant women with a single umbilical artery or other umbilical vessel abnormalities; pregnant women who could not display two free umbilical cord segments (less than two spirals per segment) or could not obtain satisfactory ultrasound images due to low amniotic fluid and other factors; pregnant women who had repeated examinations. The ethics committee of Ningbo Women and Children’s Hospital approved this study (ethics number: EC2022-M016), and the participants signed the informed consent form.

### Study methods

An attending physician with > 10 years of experience in prenatal diagnosis completed the operation after intensive training and data collection. GE Voluson E8 was used, the probe frequency was selected from 2.0 to 5.0 MHz, and the fetal examination mode late pregnancy was determined on the instrument. All cases were measured and collected by the same experimenter with the same machine. The specific steps of prenatal ultrasound examination are as follows. (1) Routine prenatal examination was performed to measure fetal size, check for fetal abnormalities, and record amniotic fluid. (2) The insertion point of the placenta into the umbilical cord was identified through careful fetal umbilical cord observation, and an abnormal insertion was recorded. Then, careful scanning was conducted along the umbilical cord to the belly button to observe umbilical cord entanglement. (3) The pitch values of two free umbilical cord segments were measured and recorded separately, and the average spiral length of the two segments was calculated. (4) The diameters of the above umbilical cord segments, the two umbilical arteries, and the umbilical vein were measured, and then, the cross-sectional area of the umbilical cord and the umbilical vessels was obtained with the area calculating method. Afterward, Wharton’s jelly area was obtained by subtracting the cross-sectional area of the umbilical vessels from that of the umbilical cord. (5) The umbilical artery blood flow systolic/diastolic (S/D) ratio, the umbilical artery resistance index (RI), and the umbilical artery pulsatility index (PI) of the above umbilical cord segments were measured and recorded, and their mean values were calculated. (6) Basic information, such as the ages of the pregnant women and gestational ages at measurement, were recorded. (7) The delivery method, cord torsion, cord edema, cord entanglement, gestational age at delivery, newborn gender, newborn weight, and conditions of amniotic fluid and placenta were followed up after delivery.

### Relevant definitions and diagnostic criteria

**Fig. 1:**
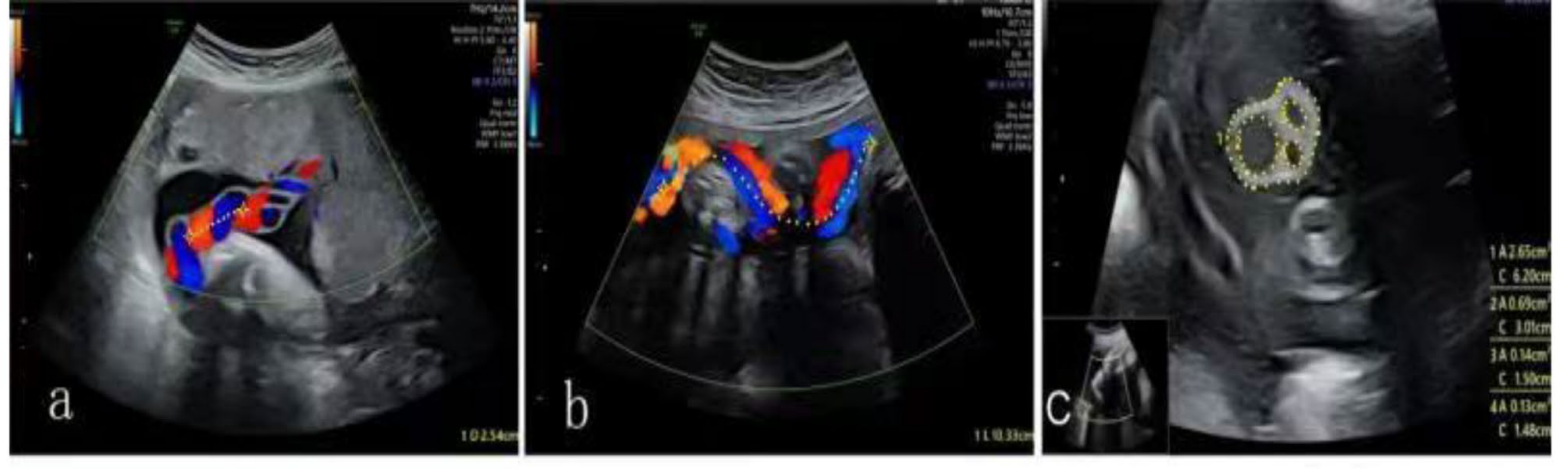
Schematic diagrams of prenatal ultrasound measurements. The yellow asterisks are the measured distance, and the yellow dotted line traces the measured trajectory. **(a)** Schematic diagram of the pitch value measurement when the umbilical cord traveled straight. **(b)** Schematic diagram of the measurement of the pitch value by the curve method through tracing along the umbilical cord when the cord traveled curved. **(c)** Schematic diagram of the measurement of Wharton’s jelly thickness

1) Definition and measurement standard of the pitch value. The pitch value is the length of one umbilical cord spiral, which is the length of a turn of umbilical cord torsion. The value is the distance from the lateral edge of a spiral umbilical vein to that of the adjacent next spiral umbilical vein according to the prenatal ultrasound measurement standard. The length of a curved umbilical cord is measured with the curve method by tracing along the umbilical cord (Fig. 1a–b).

2) Determination of Wharton’s jelly thickening. This study determined the Wharton’s jelly thickness was by Wharton’s jelly area calculated as the aforementioned method. The thickening is determined at Wharton’s jelly area greater than the 90th percentile/187.6 mm^2^ (Fig. 1c).

3) Definition of placental abnormality. Placental abnormalities include a placental thickness of > 50 mm, abnormal umbilical cord insertion into the placenta (racket placenta or sail placenta), abnormal placental position (placenta previa or low placed placenta), abnormal placental morphology (conile placenta, membranous placenta, etc.), and abnormal placental number (accessory placenta, segmented placenta, etc.), excluding abruptio placenta.

4) Determination of abnormal amniotic fluid index. Amniotic fluid index of < 50 mm indicates oligohydramnios while > 200 mm indicates polyhydramnios according to the standard of prenatal ultrasound examination.

5) Diagnosis of umbilical cord entanglement. The obstetrician can visually judge the cord entanglement after delivery. Any umbilical cord found to be entangled in any part of the fetal limbs and neck is recorded as entanglement.

6) Diagnosis of umbilical cord torsion. The cord torsion can be directly determined by the obstetrician after delivery by counting the number of umbilical cord spirals and measuring the cord length. This study defined an umbilical cord spiral index of > 0.36 coils/cm after delivery as cord torsion; for example, the condition of a 55-cm long umbilical cord with 20 spirals was defined as umbilical cord torsion [3].

7) Diagnosis of umbilical cord edema. Umbilical cord edema is determined by pathological diagnosis.

8) Newborn weight. The obstetrician or midwife obtains the newborn weight immediately after delivery.

### Statistical analysis

Statistical Package for the Social Sciences version 19.0 statistical software was applied for statistical analysis of data. The continuous quantitative data were expressed as mean ± standard deviation and percentile; the dichotomous qualitative data were expressed as a rate (%). Correlation analysis was performed using linear regression, and *P*-values of < 0.05 indicates that the two variables are significantly correlated.

## Results

### General data

This study included 528 pregnant women at the 28th week of gestation to before delivery who underwent routine prenatal ultrasound examinations in the mentioned hospital. This study excluded 10 cases of single umbilical artery, 255 cases that cannot clearly show two free umbilical cord, and 28 cases with repeated measurements. The age of the participants was 30.11 ± 4.8 years, the gestational age was 36.04 ± 2.92 weeks, the pitch value was 51.18 ± 29.13 mm, the amniotic fluid index was 121.6 ± 38.31, the UA S/D was 2.46 ± 0.48, the UA RI was 0.58 ± 0.07, the UA PI was 0.88 ± 0.19, and the fetal weight was 3265.29 ± 510.53 g. The measured pitch values in this study were positively skewed distributions with a median of 41.0 mm (Table 1).

**Table 1:** Table of percentile for quantitative data

Variables	Mean ± SD (n = 528)
Age (years)	30.11 ± 4.8
Gestation age (weeks)	36.04 ± 2.92
Pitch value (mm)	51.18 ± 29.13
Amniotic fluid index (mm)	121.6 ± 38.31
UA S/D	2.46 ± 0.48
UA RI	0.58 ± 0.07
UA PI	0.88 ± 0.19
Neonatal weight (g)	3265.29 ± 510.53

UA: umbilical artery; S/D: systolic/diastolic ratio; RI: resistance index; PI: pulsatility index

Among the 528 cases, 63 (11.9%) had Wharton’s jelly thickening, 57 (10.8%) had placental abnormalities, 197 (37.3%) had cord entanglement, 49 (9.3%) had cord torsion, and 43 (8.2%) had cord edema. The ratio of female/male for the fetal gender was 256 (48.5%)/272 (51.5%) (Table 2).

**Table 2:** Statistical description of qualitative data

Variables		
Wharton’s jelly thickness	Normal 465 (88.1%)	Thickening 63 (11.9%)
Placenta	Normal 471 (89.2%)	Abnormal 57 (10.8%)
Cord entanglement	Without 331 (62.7%)	With 197 (37.3%)
Cord torsion	Without 479 (90.7%)	With 49 (9.3%)
Cord edema	Without 485 (91.8%)	With 43 (8.2%)
Fetal gender	Female 256 (48.5%)	Male 272 (51.5%)

### Comparison between pregnancy normal group and abnormal group

The pregnancy normal group and abnormal group have no significant difference in the age of pregnant women (30.04 ± 4.79 vs. 30.16 ± 4.81 years), gestational age (36.13 ± 2.83 vs. 35.97 ± 3.01 weeks), amniotic fluid index (122.33 ± 38.53 vs. 121.10 ± 38.20), UA S/D (2.47 ± 0.49 vs. 2.45 ± 0.47), UA RI (0.58 ± 0.070 vs. 0.58 ± 0.069), UA PI (0.89 ± 0.21 vs. 0.87 ± 0.17), fetal weight (3309.09 ± 492.52 vs. 3228 ± 523.21 g), fetal gender (male/female: 119/122 vs. 153/134), and placenta (normal/abnormal: 213/28 vs. 258/29), except for pitch value (45.47 ± 24.90 vs. 55.97 ± 31.51 mm) having a significant difference (*p* = 0.001) (Table 3).

**Table 3:** Comparison between the normal and the abnormal groups

Variables	Normal group (n = 241)	Abnormal group (n = 287)	t/χ^2^	*p*
Age of pregnant woman (years)	30.04 ± 4.79	30.16 ± 4.81	-0.301	0.763
Gestation age (weeks)	36.13 ± 2.83	35.97 ± 3.01	0.640	0.522
Pitch value (mm)	45.47 ± 24.90	55.97 ± 31.51	-4.276	**< 0.001**
Amniotic fluid index	122.33 ± 38.53	121.10 ± 38.20	0.398	0.690
UA S/D	2.47 ± 0.49	2.45 ± 0.47	0.558	0.577
UA RI	0.58 ± 0.070	0.58 ± 0.069	0.555	0.579
UA PI	0.89 ± 0.21	0.87 ± 0.17	1.381	0.168
Neonatal weight (g)	3309.09 ± 492.52	3228 ± 523.21	1.820	0.069
Fetal gender			0.811	0.368
male	119	153		
female	122	134		
Placenta			0.312	0.577
normal	213	258		
abnormal	28	29		

UA: umbilical artery; S/D: systolic/diastolic ratio; RI: resistance index; PI: pulsatility index

### Linear regression analysis of the pitch values of umbilical cord

this study included 13 factors, such as the age of the pregnant woman, gestational age, umbilical cord diameter, Wharton’s jelly thickness, cord torsion, cord entanglement, cord edema, placental condition, amniotic fluid index, umbilical artery flow ratio S/D, delivery method, fetal gender, and neonatal weight, as independent variables for linear regression analysis based on clinical experience. The independent variables with a linear relationship with the dependent variable were subjected to multiple linear regression analysis. Finally, 7 variables that were significantly associated with the pitch values were obtained, including cord torsion (*p* < 0.001, 95% CI=-34.81 to -19.01), cord entanglement (*p* < 0.001, 95% CI = 10.71 to 20.11), thickening of Wharton’s jelly (*p* = 0.001, 95% CI = 5.39 to 20.24), and cord edema (*p* = 0.015, 95%CI = 2.09 to 19.44), gestational age (*p* = 0.024, 95%CI = 0.14 to 1.89), age of pregnant woman (*p* = 0.009, 95%CI= -1.15 to -0.16), and neonatal weight (*p* = 0.011, 95%CI = 0.002 to 0.012). Among them, cord torsion and age of pregnant women were negatively correlated with the pitch value, while cord entanglement, Wharton’s jelly thickening, cord edema, and gestational age were positively correlated with the pitch value (Table 4). These independent variables explained 20.0% of the variation in pitch values.

**Table 4:** Results of linear regression correlation analysis of pitch values

Variables	Univariate analysis				Multivariate analysis			
	OR value	*p*	95% CI		adjusted OR (aOR) value	*p*	95% CI of aOR	
Age	-0.66	**0.013**	-1.17	-0.14	-0.66	**0.009**	-1.15	-0.16
Gestation age	0.95	**0.028**	0.11	1.80	1.01	**0.024**	0.14	1.89
Diameter of umbilical cord	-0.33	0.62	-1.62	0.96	-1.06	0.100	-2.32	0.21
Thickening of Wharton’s jelly	13.43	**0.001**	5.83	21.03	12.82	**0.001**	5.39	20.24
Amniotic fluid index	-0.08	**0.021**	-0.14	-0.01	-0.046	0.144	-0.11	0.02
UA S/D	2.62	0.32	-2.58	7.82	3.54	0.170	-1.51	8.59
UA RI	21.89	0.230	-13.92	57.71	40.03	0.501	-76.70	156.77
UA PI	11.17	0.096	-1.99	24.34	25.05	0.069	-1.93	52.03
Abnormal placenta	-3.68	0.37	-11.71	4.35	-4.04	0.281	-11.40	3.32
Cord torsion	-29.51	**< 0.001**	-37.72	-21.30	-26.91	**< 0.001**	-34.81	-19.01
Cord edema	9.40	**0.042**	0.32	18.48	10.77	**0.015**	2.09	19.44
Cord entanglement	16.03	**< 0.001**	11.06	20.99	15.41	**< 0.001**	10.71	20.11
Cesarean delivery	1.41	0.581	-3.60	6.41	4.33	0.072	-0.39	9.05
Fetal gender	0.99	0.695	−3.99	5.98	0.34	0.885	−4.27	4.95
Neonatal weight	0.87	0.72	−3.94	5.67	0.007	**0.011**	0.002	0.012

OR: overall rate; aOR: adjusted overall rate; CI: confidence interval; UA: umbilical artery; S/D: systolic/diastolic ratio; RI: resistance index; PI: pulsatility index

## Discussion

Several studies [4–6] have recently established a link between abnormal umbilical cord spiral and a variety of adverse perinatal outcomes. The length of an umbilical cord spiral, or the umbilical cord pitch value, can be determined by measuring a segment that is visible in an ultrasonogram. The pitch value indicates the number of umbilical cord spirals to some extent without witnessing the entire cord, but its accuracy remains being explored in research [7]. A multivariate regression analysis of the spiral values in this study revealed cord torsion, cord entanglement, Wharton’s jelly thickening, cord edema, gestational age, age of pregnant woman, and neonatal weight as the significant correlates. A tight cord spiral is highly associated with cord torsion, according to several earlier studies. However, there is less evidence linking tight cord spirals to other cord anomalies, such as cord entanglement, cord edema, and Wharton’s jelly thickening.

This study revealed that cord wrapping around the neck or the body had a substantial impact on the size of the umbilical cord spiral. Additionally, the pitch value significantly increased in the part of the cord that wrapped around the fetal limb. Hence, the spiral was not evenly distributed throughout the cord and became more sparse. A study on postnatal umbilical cord spiral structure by Strong et al., in 1996 [8], revealed significantly fewer umbilical cord spirals in fetuses with cord entanglement than those without cord entanglement, which is in line with our current findings. However, no additional pertinent research has been published since Liu Tianxin et al. [9] reported, in 2011, that cord entanglement could increase the umbilical cord pitch value.

The pitch value was closely related to cord torsion and entanglement in this study, as well as to cord edema and Wharton’s jelly thickening, which has only rarely been documented in earlier research. Several earlier studies have noted the association between sparse umbilical cord spirals and various adverse perinatal outcomes [10–12], but no study has highlighted Wharton’s jelly thickening as one of the potential reasons for sparse umbilical cord spirals. Our study revealed a negative correlation between the umbilical cord pitch value and the age of pregnant women. Further, the older the mother, the lower the pitch values and the tighter the umbilical cord spirals. However, further studies are needed to verify these results. A significant correlation was also discovered between the pitch values and gestational ages, but the correlation coefficient was not high, which is similar to a previous study [13]. The cord pitch values remained varied between the mid- and late-trimester, with the late-trimester values being higher, although several earlier studies reported that the cord length gradually increased after 20 weeks. The present study revealed that the gestational age ranged from 28 weeks to the day before delivery, and the spiral values during this period remained affected by the gestational age, which was positively correlated with the gestational age. The lengthening of gestational ages increased the pitch values but to a lesser extent. Neonatal weight was likewise favorably correlated with pitch values while a substantial association was found between weight and gestational age. Older fetuses have longer umbilical cords, while heavier fetuses are likely to have longer umbilical cords. However, further research is required to confirm this discovery by examining correlations between neonatal weight and pitch values at the same gestational age.

This study revealed no significant correlation between the pitch value and placenta, cord diameter, umbilical artery flow ratio, and amniotic fluid index in the multivariate regression analysis. The amniotic fluid index, which is a significant correlate of the pitch value in the univariate analysis, was negatively correlated with the pitch value (with a low correlation coefficient), indicating that the correlation between the amniotic fluid index and pitch value may be small and affected by confounding factors. However, no significant correlation was found between spiral values and umbilical artery flow parameters in either univariate or multivariate analysis. Multiple earlier investigations on cord spiral abnormalities and umbilical blood flow [14–16] revealed that a tight cord spiral is associated with increased blood flow in the umbilical vein at the umbilical foramen. Currently, no agreed-upon conclusion exists about the connection between the umbilical artery blood flow and the cord spiral. In line with the findings of Predanic et al. [14], our study indicated no significant correlation between the cord spiral and umbilical artery blood flow.

Our findings indicate that pitch values could be useful in a routine clinical setting. First, we can screen fetuses with excessive cord torsion by assessing umbilical cord pitch values and performing closer prenatal monitoring. We need to carefully monitor the umbilical cord at the umbilical chakra (where the cord enters the fetal abdomen) for excessive twisting in such fetuses that could lead to umbilical vessel occlusion or even rupture, to choose the appropriate time to terminate the pregnancy and avoid the risk of intrauterine fetal death. Additionally, umbilical cord pitch value assessment can help indicate the presence of umbilical cord entanglement (mainly umbilical cord entanglement of the limb, which is often difficult to determine by prenatal ultrasound) and assess the tightness of umbilical cord entanglement. Pitch value measurement can be used to screen for fetuses with tightly wrapped umbilical cords in late pregnancy, which can provide closer monitoring of such fetuses and useful information in choosing the mode of delivery, such as if the tightly wrapped fetus is not suitable for normal delivery or requires closer monitoring during normal delivery, which will be of great clinical help.

Some restrictions and perspectives of the present study are as follows: (i) the present study was a descriptive study, without study subject groupings. The correlation between the independent and dependent variables was only initially explored, which needs to be further confirmed by cohort or pilot studies. (ii) Other possible factors associated with the pitch values were not included in this study, such as maternal factors and umbilical cord knotting, and more factors can be included in regression analysis to determine their link with the pitch value in the future. (iii) Prenatal ultrasound has its limits when it comes to studying umbilical cord morphology and displaying a large pitch value in a small amniotic fluid gap is challenging; therefore, the final selection of cases with small umbilical cord pitch values was more biased. Accordingly, future morphological examinations of the umbilical cord may be more accurately performed using other imaging techniques, such as magnetic resonance imaging.

## Conclusion

Overall, cord entanglement, cord torsion, cord edema, Wharton’s jelly thickening, age of pregnant women, gestational age, and neonatal weight are significantly associated with the pitch value after initially investigating the factors associated with umbilical cord pitch value. However, further cohort studies or pilot studies are needed to explore the causal relationships.

## Data Availability

The dataset generated and/or analysed during the study are available from the corresponding author on reasonable request.
